# A Novel Time Series Approach to Bridge Coding Changes with a Consistent Solution Across Causes of Death

**DOI:** 10.1007/s10680-013-9307-4

**Published:** 2014-06-07

**Authors:** Ronald H. M. van der Stegen, L. G. H. Koren, Peter P. M. Harteloh, Jan W. P. F. Kardaun, Fanny Janssen

**Affiliations:** 1 Methodology Department, Statistics Netherlands, PO Box 24500, 2490 HA The Hague, The Netherlands; 2 Health and Care Department, Statistics Netherlands, PO Box 24500, 2490 HA The Hague, The Netherlands; 3 Population Research Centre, Faculty of Spatial Sciences and Unit of PharmacoEpidemiology & PharmacoEconomics, Pharmacy Department, University of Groningen, PO Box 800, 9700 AV Groningen, The Netherlands

**Keywords:** Time series analysis, Cause-specific mortality, ICD revision, Coding change

## Abstract

Revisions of the International Classification of Diseases (ICD) can lead to biases in cause-specific mortality levels and trends. We propose a novel time series approach to bridge ICD coding changes which provides a consistent solution across causes of death. Using a state space model with interventions, we performed time series analysis to cause-proportional mortality for ICD9 and ICD10 in the Netherlands (1979–2010), Canada (1979–2007) and Italy (1990–2007) on chapter level. A constraint was used to keep the sum of cause-specific interventions zero. Comparability ratios (CRs) were estimated and compared to existing bridge coding CRs for Italy and Canada. A significant ICD9 to ICD10 transition occurred among 13 cause of death groups in Italy, 7 in Canada and 3 in the Netherlands. Without the constraint, all-cause mortality after the classification change would be overestimated by 0.4 % (NL), 0.03 % (Canada) and 0.2 % (Italy). The time series CRs were in the same direction as the bridge coding CRs but deviated more from 1. A smooth corrected trend over the ICD-transition resulted from applying the time series approach. Comparing the time series CRs for Italy (2003), Canada (1999) and the Netherlands (1995) revealed interesting commonalities and differences. We demonstrated the importance of adding the constraint, the validity of our methodology and its advantages above earlier methods. Applying the method to more specific causes of death and integrating medical content to a larger extent is advocated.

## Introduction

The study of cause-specific mortality levels and trends is very relevant for monitoring the health situation of countries, and for the underlying patterns. However, changes in cause-specific mortality reflect not only real changes in mortality due to medical treatment, life style changes, environmental changes, etc., but can also stem from changes in classification, i.e. the assignment of codes to the underlying cause of death reported on the death certificate. The most important are the changes in coding that stem from official revisions of the International Classification of Diseases (ICD). Since its initiation in 1893, this international standard for the coding of causes of death has been revised ten times in the twentieth century (WHO 2004; Anderson 2011). These ICD revisions are necessary and unavoidable, to keep the classification in pace with the developments in medical knowledge and medical technology. In an ideal world, there should be no other coding changes of causes of death, but in reality every year changes in data collection, processing and coding can occur, most of them minor. Still however, all these coding changes can result in serious bias in cause-specific death numbers and breaks in cause-specific mortality trends (e.g. Anderson 2011; Janssen and Kunst 2004; Rey et al. 2011).

There are several methods to detect and to correct coding changes in long time series of deaths, most of them focussing on ICD revisions. They can be distinguished principally in dual and single coding methods, i.e. whether the *same* cases have been coded in both ICD revisions, or whether *similar* cases have been coded in both revisions.

In dual coding, mostly called ‘bridge coding’, death records for a single year (mostly a sample) are coded according to both the former and the new ICD (e.g. Anderson 2011), creating a direct link between the two classifications. However, this approach has not been implemented in many countries (see Anderson 2011; Janssen and Kunst 2004).

In the absence of dual coding, the approach by Vallin and Meslé (1988) and Meslé and Vallin (1996) is currently being adopted more and more, e.g. next to France (Meslé and Vallin 2011), in West Germany (Pechholdova 2008), the Netherlands (Wolleswinkel-van den Bosch et al. 1996) and Sweden (Statistics Sweden 1990). Their approach involves the construction of concordance tables, linking the items in two successive ICD revisions based on medical content and the calculation of transition ratios through the cross tabulation of death numbers for the first year of the new ICD according to the codes of the former ICD (Vallin and Meslé 1988).

Both aforementioned approaches are very costly and labour intensive (Meslé and Vallin 2008). They can only be applied to one country at a time and only take into account data for a single year or for two subsequent years, ignoring normal year-to-year fluctuations. To overcome these issues time series approaches were introduced recently, where a longer series of data is considered and ‘normal’ annual fluctuation is distinguished from the ‘special’ event due to revision of the classification (see intervention analysis in for instance Chatfield (2004)). For example, Janssen and Kunst (2004) detected and corrected for mortality jumps caused by coding changes both between and within ICD revisions using a log-linear regression approach and visual inspection of the trends. They applied their approach in several international public health studies (Janssen et al. 2004, 2005; Janssen and Kunst 2005).

Rey et al. (2011) expanded on this methodology by using an automatic jump detection method instead of the visual detection of jumps or a priori selection of years in which the jumps are likely to occur.

An aspect that has been ignored in these time series approaches, however, is that the procedure should result in a consistent solution across causes of death. The total number of deaths in a year should not change if a revised classification is introduced. So, if a certain number of deaths are removed from a certain time series because of coding changes, these should be added to another time series.

The objective of our study is to present a time series approach which provides a consistent solution across causes of death, i.e. the total number of death over all causes in a year is preserved. We apply our approach to Canada, Italy and the Netherlands and compare our method with the existing bridge coding approach for the ICD9–ICD10 transition for Italy and Canada.

The ICD9–ICD10 transition is regarded as the most rigorous since decades. More detail was added as well as newly recognised diseases, leading to an enormous increase of codes from ~6,000 in the ICD9 revision to ~10,000 in the ICD10 revision. In addition, some diseases and groups of conditions have been moved from one ICD chapter to another in line with new insights on aetiology and pathology. At the same time, considerable changes to the rules governing the selection of the underlying cause were implemented resulting in more explicit but complex instructions (Anderson et al. 2001; see WHO 1992; ONS 2012a, b, c; de Boo et al. 1998) for more information). Previous attempts at bridging the two coding schemes showed indeed lost continuity. Examples can be found in Meslé and Vallin (2008), Geran et al. (2005), Pace et al. (2007), Pechholdova (2008), ISTAT (2011), Rooney et al. (2002), Janssen and Kunst (2004), Rey et al. (2011) and used the different methods that were described and discussed above, i.e. bridge coding, approach by Meslé and Vallin and time series approach. In none of the previous time series approaches a consistent solution across causes of death was safeguarded.

## Data and Methods

For Italy, Canada and the Netherlands, we obtained data on the numbers of death by cause and year for ICD9 and ICD10 for both sexes and all ages combined. See Table 1 for the years to which the ICD9 and the ICD10 apply in these countries (ISTAT 2011; Geran et al. 2005; Sonsbeek 2005). ICD 10 was first adopted in the Netherlands (1996), 4 years later in Canada and again 3 years later in Italy.

**Table 1:** Years to which the ICD9 and ICD10 apply in Italy, Canada and the Netherlands

Country	ICD9		ICD10		Double coding/CRs	Remarks
	From	Until	From	Until		
Canada	1979	1999	2000	2007	Double coding for 1999	Manual and automatic coding
Italy	1980	2002	2003	2007	Double coding for 2003	No data for 2004 and 2005. Automatic coding except for AIDS in ICD9
The Netherlands	1979	1995	1996	2010	No double coding. CRs for the year 1995	Only manual coding

*Source information* Geran et al. (2005), ISTAT (2011), Sonsbeek (2005)

In the Italian data a transition occurred between 1989 and 1990 resulting in a jump in several causes of death. Therefore the Italian data from 1980 up until 1989 are not used in our analysis.

We distinguished 17 groups of causes of death, following the original ICD-10 Chapters (WHO 1992), except that we combined Chapters VI–VIII and ignored Chapters XIX and XXI as these were not used for coding the underlying cause of death. These same groups of causes of death were used in the bridge coding studies for Canada (Geran et al. 2005) and Italy (Pace et al. 2007; ISTAT 2011). See Table 2 for the cause of death groups we distinguished with their respective codes for ICD9 and ICD10. For the Netherlands, we decided to use the same concordance table as Italy did, which is slightly different from the concordance table used in Canada. One of the two differences relates to the classification of ‘other specified disorders involving the immune mechanism’ (279.8) in either Chapter I or Chapter III in ICD 9.

**Table 2:** The 17 causes of death groups we distinguished with their respective codes for ICD9 and ICD10 and their share in overall mortality over the applied period, Italy, Canada and the Netherlands

				Cause-proportional mortality (%)		
	ICD9–NL + Italy	ICD9–Canada	ICD10	Canada (1979–2007)	Italy (1990–2007)	NL (1979–2010)
Ch I: Certain infectious diseases and parasitic diseases	001–139 + 279.8	001–139	A00–B99	1.2	1.0	1.0
Ch II: Neoplasms	140–239	140–239	C00–D48	27.5	28.3	28.4
Ch III: Diseases of the blood and blood-forming organs and certain disorders involving the immune mechanism	279–289 excl 279.8	280–289	D50–D89	0.4	0.4	0.3
Ch IV: Endocrine, nutritional and metabolic diseases	240–278	240–279	E00–E90	3.2	3.7	2.9
Ch V: Mental and behavioural disorders	290–319	290–319	F00–F99	2.1	1.4	2.6
Ch VI–VIII: Diseases of the nervous system and sense organs	320–389	320–389	G00–H95	2.9	2.4	2.2
Ch IX: Diseases of the circulatory system	390–459	390–459	I00–I99	38.7	42.7	37.8
Ch X: Diseases of the respiratory system	460–519	460–519	J00–J99	8.2	6.3	8.7
Ch XI: Diseases of the digestive system	520–579	520–579	K00–K93	3.8	4.7	3.7
Ch XII: Diseases of the skin and subcutaneous tissue	680–709	680–709	L00–L99	0.1	0.1	0.3
Ch XIII: Diseases of the musculoskeletal system and connective tissue	710–739	710–739	M00–M99	0.5	0.4	0.6
Ch XIV: Diseases of the genitourinary system	580–629	580–629	N00–N99	1.7	1.4	2.1
Ch XV: Pregnancy, childbirth and the puerperium	630–676	630–676	O00–O99	0.009	0.004	0.01
Ch XVI: Certain conditions originating in the perinatal period	760–779	760–779 excl 771.3	P00–P99	0.6	0.3	0.4
Ch XVII: Congenital malformations deformations and chromosomal abnormalities	740–759	740–759	Q00–Q99	0.6	0.3	0.5
Ch XVIII: Symptoms, signs and abnormal clinical and laboratory finding not elsewhere classified	780–799	780–799	R00–R99	1.4	1.6	4.2
Ch XX: External causes of morbidity and mortality	E800–E999	E800–E999	V01–Y98	7.0	4.9	4.2
All-cause mortality (death numbers 2007)	001–E999	001–E999	A00–Y98	235,217	572,881	133,022

*Data source* Canada: ICD 9: Nadine Ouellette, Statistics Canada: ICD 10 http://www.statcan.gc.ca/pub/84-208-x/2012001/tbl-eng.htm, ISTAT, Italy: http://timeseries.istat.it/fileadmin/allegati/Sanita/tavole_inglese/Table_4.9.1.xls, Statistics Netherlands: http://statline.cbs.nl/StatWeb/selection/?DM=SLEN&PA=7052ENG&LA=EN&VW=T

*Chapter definitions* Geran et al. (2005), ISTAT (2011)

Using the respective codes for ICD9 and ICD10, we performed time series analysis to cause-proportional mortality for all ages and both sexes combined for Italy (1990–2007), Canada (1979–2007) and the Netherlands (1979–2010) through

1 $$ x_{j,t} = y_{j,t} + i_{j,t} + b_{j} \delta_{t} , $$

where *x*_*j,t*_ is share of deaths from cause *j* in all-cause mortality in year *t*, *y*_*j,t*_ is the annual time trend for cause *j* devoid of the annual fluctuation, *i*_*j,t*_ is the irregular component of the time series for cause *j*, with average 0, reflecting annual fluctuation, *b*_*j*,_ is the intervention, i.e. the estimated jump due to the ICD9–ICD10 transition for cause *j*, and *δ*_*t*_ = 1 for ICD10 (Canada ≥ 2000, Italy ≥ 2003, the Netherlands ≥ 1996) and 0 for ICD9 (the intervention is equal in magnitude but opposite of sign when 0 and 1 are reversed).

To make sure that the sum of the repaired series is equal to the total number of deaths during the ICD9–ICD10 transition, we added the constraint that the sum of the interventions must be zero, i.e.

2 $$ \sum\limits_{j} {b_{j} = 0} . $$

In this way, a consistent solution across all causes of death is obtained.

We applied our time series analysis to cause-proportional mortality as this reduces the fluctuations around the trend compared to using mortality numbers. That is, the total number of deaths (the denominator) often has more or less comparable fluctuations as the specific causes of death. In Table 2, the average cause-proportional mortality—calculated over the whole period—is provided for the three countries.

Numerous time series models exist that can solve Eqs. 1 and 2 separately. See for an overview for example Chatfield (2004). However, solving Eqs. 1 and 2 simultaneously is preferred because it reduces the number of degrees of freedom in the model. This increases the accuracy compared to solving Eq. 1 alone or Eqs. 1 and 2 separately.

Special software is required for solving Eqs. 1 and 2 simultaneously in large time series problems. As to our knowledge SsfPack is the only candidate (van den Brakel et al. 2008; Koopman et al. 2008). Ssfpack calculates the most probable solution for interventions incorporating all data of the 17 time series. The time series can be modelled with stochastic time series models such as Arima models or state space models. We opted for a local linear level and slope model with intervention in state space formulation (Commandeur and Koopman 2007) instead of an Arima model, because our choice does not require user intervention, as Arima does. The model fits the trend as a linear equation with slowly varying coefficients. The software determines the year-to-year change of the coefficients, and assigns changes larger than a certain value as a fluctuation or a jump (intervention).

The following set of equations is solved:

$$ \begin{gathered} x_{j,t} = \mu_{j,t} + b_{j} \delta_{t} + \varepsilon_{j,t} , \hfill \\ \mu_{j,t + 1} = \mu_{j,t} + \upsilon_{j,t} + \xi_{j,t} , \hfill \\ \upsilon_{j,t + 1} = \upsilon_{j,t} + \zeta_{j,t} , \hfill \\ b_{j,t + 1} = b_{j,t} = b_{j} . \hfill \\ \end{gathered} $$

With *μ*_*j,t*_ for the level and *υ*_*j,t*_ for the slope. *ε*_*j,t*_ *, ζ*_*j,t*_ and *ξ*_*j,t*_ are disturbances given by respectively NID(0, *σ*_*ε*_^2^), NID(0, *σ*_*ζ*_^2^) and NID(0, *σ*_*ξ*_^2^) (i.e. zero mean and a variance of *σ* ^2^). The first equation is called the observation or measurement equation and the second till fourth the state equations (Commandeur and Koopman 2007). For convenience, this set of equations is rewritten in state space formulation in which the equations describing one cause of death are replaced by a matrix model for the entire set of causing of death modelling equations:

$$ \begin{gathered} x_{t} = Z_{t}^{'} \alpha_{t} + \varepsilon_{t} \hfill \\ \alpha_{t + 1} = T_{t} \alpha_{t} + R_{t} \eta_{t}. \hfill \\ \end{gathered} $$

With *x*_*t*_ and *α*_*t*_ as vectors for all *j* over *x*_*j,t*_ and (*μ*_*j,t*_ *, υ*_*j,t*_ *, b*_*j,t*_) respectively. Matrices *Z*_*t*_, *T*_*t*_ and *R*_*t*_ consist of ones and zeros to represent the equations above including Eq. 2 and *η*_*t*_ represents all disturbances of the state equations.

By simultaneously estimating all different equations we use all the relevant information in a balanced manner and therefore obtain a more accurate result than other—more simple—mathematical techniques, such as the rule of three or a Lagrange optimisation.

The significance of the intervention due to the ICD9–ICD10 transition is assessed by its standard deviation, stdev(*b*_*j*_,). Using a 95 % confidence interval a significant break occurs when 1.96 × stdev(*b*_*j*_,) ≤ |*b*_*j*_|.

In addition, we estimated comparability ratios (CRs), as first derived by Erhardt and Werner (1950), which in bridge coding represent the proportion of cause-specific deaths coded according to the new ICD revision divided by the cause-specific deaths according to the former ICD revision (Anderson 2011). We did so through

3 $$ {\text{CR}}_{j,9 \to 10} = \frac{{y_{j,t} + b_{j} }}{{y_{j,t} }}, $$

where the intervention *b*_*j*_ represents the difference in cause-specific mortality between ICD10 and ICD9. We used the trend *y*_*j,t*_ instead of the real value *x*_*j,t*_ as to prevent that the CR is influenced by coincidental fluctuations (Anderson 2011). The confidence interval of the CR, presented in Eq. 3, is not calculated because of the unknown cross-correlations between the trend *y*_*j,t*_ and the intervention *b*_*j*_.

For Canada and Italy, the time series CRs were compared to the existing bridge coding CRs (Geran et al. 2005; Pace et al. 2007; ISTAT 2011). A comparison of the confidence intervals was regarded not meaningful, because both CRs take different effects into account—i.e. analysis of individual records in 1 year for bridge coding versus time dependent analysis of aggregates for time series analysis. Besides, the confidence interval for bridge coding often only includes the survey error, whereas this is not the only error in bridge coding studies. For instance, manual coding of deaths certificates is not 100 % repeatable with the same result for the same ICD, as has been shown by Harteloh et al. (2010). In case of automatic coding, certain cases will be rejected introducing a potential bias. Also, in automatic coding about 20 % of cases are actually coded using some manual assistance (Pavillon et al. 1998).

## Results

Our time series analysis reveals statistically significant transitions (at 95 % confidence interval) from ICD9 to ICD10 for 13 out of 17 cause of death groups in Italy (Table 3). For Canada and the Netherlands significant transitions occurred in less cause of death groups, i.e. 7 and 3, respectively. A significant transition most likely results from a high amount of discontinuity as a result of the ICD9 to ICD10 revision. The chances of a transition to become statistically significant increase also, when more deaths are involved and when coding changes within an ICD revision are minimal, as this may result in smoother time series and less variance.

**Table 3:** CR and the intervention and its standard deviation for the ICD9–ICD10 transition based on our time series analysis for 17 main groups of causes of death in Italy, Canada and the Netherlands

	Canada (1979–2007)			Italy (1979–2007)			The Netherlands (1979–2010)		
	CR	*b*_*j*_ %	stdev(*b*_*j*_) %	CR	*b*_*j*_ %	stdev(*b*_*j*_) %	CR	*b*_*j*_ %	stdev(*b*_*j*_) %
Ch I: Certain infectious diseases and parasitic diseases	1.22	**0.256**	0.098	1.26	**0.256**	0.090	0.92	−0.091	0.051
Ch II: Neoplasms	1.03	**0.883**	0.230	0.96	−**1.197**	0.382	1.01	0.239	0.499
Ch III: Diseases of the blood and blood-forming organs and certain disorders involving the immune mechanism	1.07	0.025	0.022	0.89	−**0.053**	0.017	0.54	−**0.212**	0.027
Ch IV: Endocrine, nutritional and metabolic diseases	1.06	0.211	0.116	1.10	**0.387**	0.184	1.01	0.039	0.219
Ch V: Mental and behavioural disorders	0.86	−**0.419**	0.166	0.85	−**0.266**	0.045	−	−	0.197
Ch VI–VIII: Diseases of the nervous system and sense organs	1.43	**1.325**	0.078	1.25	**0.665**	0.042	1.44	**0.676**	0.139
Ch IX: Diseases of the circulatory system	0.99	−0.393	0.287	0.98	−**0.849**	0.343	0.98	−0.711	0.452
Ch X: Diseases of the respiratory system	0.78	−**2.151**	0.236	1.1	0.615	0.318	1.06	0.504	0.406
Ch XI: Diseases of the digestive system	1.03	0.091	0.084	0.94	−**0.273**	0.113	0.99	−0.044	0.103
Ch XII: Diseases of the skin and subcutaneous tissue	1.16	**0.019**	0.009	1.13	0.017	0.012	0.91	−0.036	0.035
Ch XIII: diseases of the musculoskeletal system and connective tissue	1.39	**0.180**	0.019	1.45	**0.162**	0.014	1.12	0.069	0.047
Ch XIV: Diseases of the genitourinary system	1.02	0.038	0.031	1.07	**0.100**	0.041	0.96	−0.083	0.098
Ch XV: Pregnancy, childbirth and the puerperium	1.20	0.001	0.003	2.28	0.002	0.002	1.67	**0.006**	0.002
Ch XVI: Certain conditions originating in the perinatal period	1.14	0.053	0.033	0.90	−0.023	0.018	0.98	−0.008	0.028
Ch XVII: Congenital malformations deformations and chromosomal abnormalities	1.01	0.005	0.028	1.25	**0.060**	0.013	0.98	−0.009	0.016
Ch XVIII: Symptoms, signs and abnormal clinical and laboratory finding, not elsewhere classified	1.05	0.068	0.247	1.58	**0.665**	0.101	1.01	0.055	0.420
Ch XX: External causes of morbidity and mortality	0.97	−0.193	0.197	0.94	−**0.268**	0.108	1.00	−0.017	0.131
Ch I–XX: All deaths	1.00	0.000	0.000	1.00	0.000	0.000	1.00	0.000	0.000

Bold numbers indicate significant breaks according to the 95 % confidence interval. The time series were derived according to the specific ICD codes given in Table 2. The intervention *b*_*j*_ and its standard deviation stdev(*b*_*j*_) are expressed in percentages because we modelled cause-proportional mortality

**Fig. 1:**
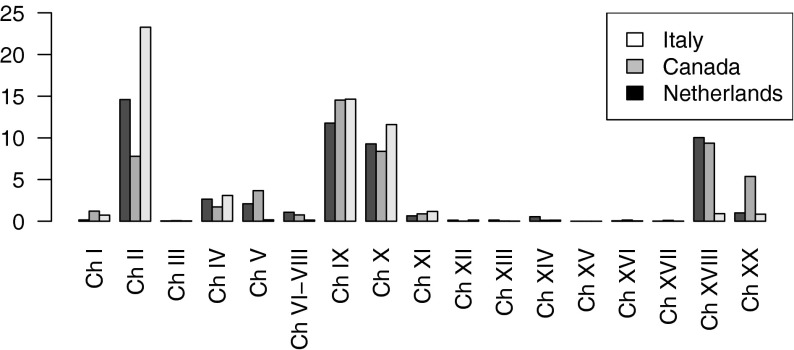
The percentage reduction in the standard deviation of the interventions by adding the constraint to the model. *Note* the cause of death groups to which the Chapter numbers of the *x*-axis refer to can be found in Table 2

The last row in Table 3 shows the consistency over all causes of death of our approach. Solving the time series model without the constraint that the sum of the cause-specific interventions should be zero, resulted in an increase of the all-cause mortality rate after the classification change of 0.4 % (=516 deaths) for the Netherlands, 0.03 % (=35 deaths) for Canada and 0.2 % (=1,153 deaths) for Italy. This is an essential increase, especially because the cause-specific transitions are of the same order of magnitude. In Fig. 1 the effect of adding the constraint to the standard deviation of the interventions is shown. For some interventions, the percentage reduction in the standard deviation is negligible, while for others the reduction is high. Comparing the percentage reduction in Fig. 1 with the magnitude of the standard deviation in Table 3, it follows that the large standard deviations are reduced more than the smaller ones.

**Fig. 2:**
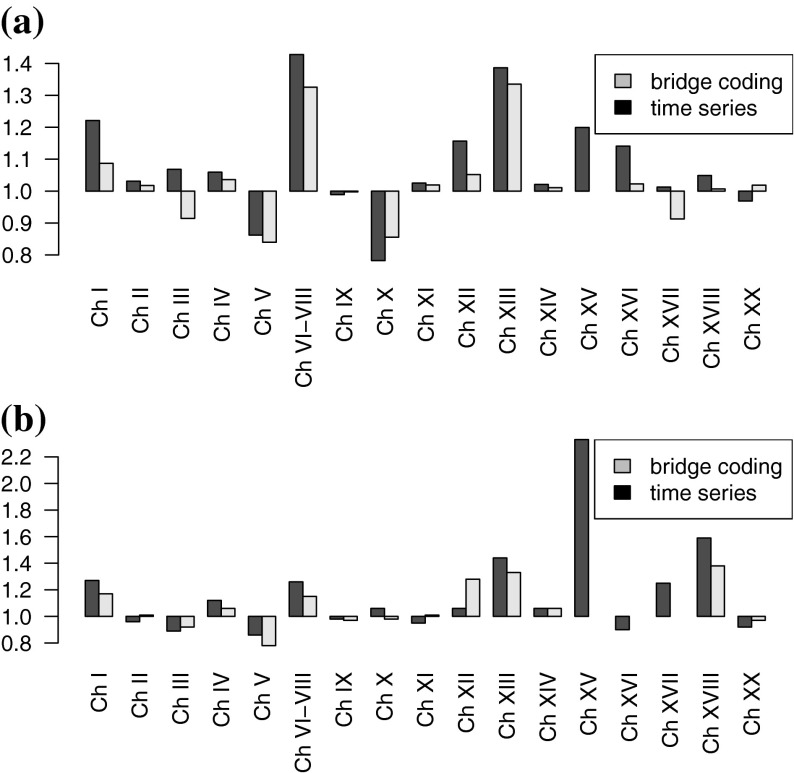
Comparison of the CRs by bridge coding and time series analysis for the ICD9–ICD10 transition. Canada (1999) (**a**) and Italy (2003) (**b**). *Note* for Chapter *XV* no bridge coding CR existed in Canada and Chapter *XV*, *XVI* and *XVII* no bridge coding CR existed in Italy. The cause of death groups to which the Chapter numbers of the *x*-axis refer to can be found in Table 2

Comparing the CRs for the ICD9–ICD10 transition estimated by time series analysis with the existing bridge coding CRs in Canada and Italy showed equal directions but differences in magnitude with the time series CR generally being more extreme (Fig. 2). This is surprising considering that the time series approach averages yearly fluctuations. For Canada, in 12 out of 16 cause of death group comparisons the CRs have the same direction while the time series CRs are more extreme. The same applied to 11 comparisons for Italy.

For Chapter XV (Pregnancy, childbirth and the puerperium) in Canada and Italy, Chapter XVI (Certain conditions originating in the perinatal period) in Italy and Chapter XVII (Congenital malformations deformations and chromosomal abnormalities) in Italy, bridge coding CRs do not exist, whereas they can be estimated by time series analysis.

For Canada for Chapters III (Diseases of the blood and blood-forming organs and certain disorders involving the immune mechanism), XVII (Congenital malformations deformations and chromosomal abnormalities) and XX (External causes of morbidity and mortality) and for Italy for Chapters II (Neoplasms), X (Diseases of the respiratory system), and XI (Diseases of the digestive system), the direction of the CR was different for the two approaches. In all these instances the CRs were close to 1, which often coincides with a possible inaccurate determination of the intervention.

**Fig. 3:**
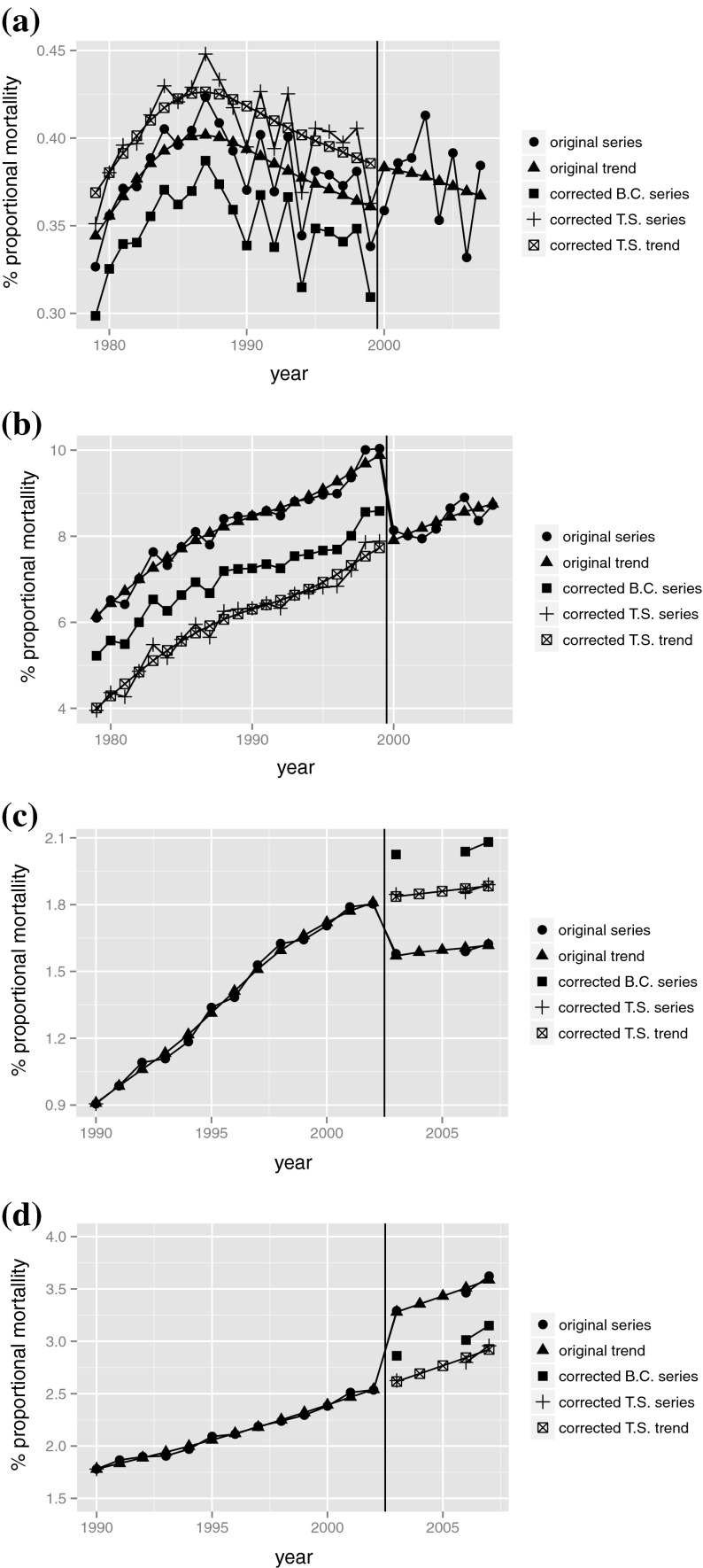
Examples of outcomes of the time series approach including the comparison of corrected series of cause-proportional mortality (%) based on the time series approach (T.S.) with those based on bridge coding (B.C.). Note that the Italian data for the years 2004 and 2005 is non-existent and therefore missing in the original series and in the corrected B.C. series. **a** Canada, Chapter III: diseases of the blood and blood-forming organs and certain disorders involving the immune mechanism. **b** Canada, Chapter X: Diseases of the respiratory system. **c** Italy, Chapter V: Mental and behavioural disorders. **d** Italy, Chapter VI–VIII: Diseases of the nervous system and sense organs

Figure 3 shows four examples of our time series approach versus bridge coding. For both Chapter X (Diseases of the respiratory system) in Canada (a) and Chapter VI–VIII (Diseases of the nervous system and sense organs) in Italy (b) the time series CR were more extreme than the bridge coding CR. Chapter III (Diseases of the blood and blood-forming organs and certain disorders involving the immune mechanism) for Canada (c) and Chapter V (Mental and behavioural disorders) for Italy (d) revealed opposite CRs.

The figures show the original time series, the original trend, the trend and the time series corrected for the coding changes using our time series approach and the time series corrected for the coding changes using bridge coding. The vertical line denotes the transition from ICD 9 to ICD 10. Italy—in its bridge coding—calculated ICD9 from ICD 10 and therefore the correction is calculated for the period before the transition to ICD10. Canada, on the opposite, calculated ICD 10 from ICD 9 and therefore the correction is calculated for the period after the transition.

The model calculates in all cases a smooth original trend, except in the transition. When—in the corrected time series trend—the intervention is added to the trend, a smooth continuation of the trend over the classification change shows, as is assumed by the model. We compare the corrected cause-proportional mortality (%) based on our time series approach with the corrected series calculated using the bridge coding CR. In all four instances, our corrected series is close to the corrected trend, while the bridge coding series clearly is not.

The corrected trend and series displayed in these figures do not necessarily represent the optimal way of reconstructing long time series. See as well Sect. 4 paragraph 9.

**Fig. 4:**
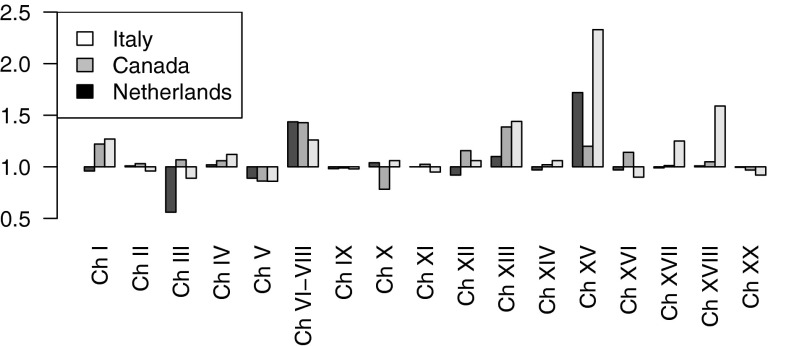
Comparison of the time series CRs for the ICD9–ICD10 transition in Canada (1999), Italy (1995) and the Netherlands (2003). The cause of death groups to which the Chapter numbers of the *x*-axis refer to can be found in Table 2

The time series CRs for Italy (2003), Canada (1999) and the Netherlands (1995) were in similar directions for all three countries for Chapters IV, V, VI–VIII, IX, XIII, XV, and XVIII (Fig. 4; Table 3). Large positive CRs showed for diseases of the nervous system (Chapter VI–VIII), diseases of the musculoskeletal system (Chapter XIII) (not NL), and diseases related to pregnancy, childbirth and the puerperium (Chapter XV). Large negative CRs were observed for mental and behavioural disorders (Chapter V). The time series CRs for Italy were larger than or equal to those for Canada and the Netherlands for 14 cause of death groups. For Italy, the CRs were, however, mostly in the same direction as (one of) the other countries. For the Netherlands, the CRs were most often opposite to those in Italy and Canada (*N* = 4), for example for infectious diseases (Chapter I) and diseases of the skin (Chapter XII).

## Discussion

### Reflection on the Methodology

In this paper, we presented a time series approach to bridge ICD coding changes in cause-specific mortality trends. As an initial step we applied our approach to 17 cause of death groups. Our approach includes an important property above other time series approaches in that a consistent solution across causes of death is obtained. We did so by setting a constraint on interventions modelling the ICD-transition imposing that the sum of the interventions must be zero, i.e. the total number of deaths is the same before and after the removing interventions. Our results showed that the effect of this constraint can be significant, with a decline in all-cause mortality of 0.4 % (NL), 0.03 % (Canada) and 0.2 % (Italy). Because of the constraint, our approach can accurately be applied to all the deaths in a country, which is an additional advantage over the time series approach by Rey et al. (2011) which was only applied to a selection of causes of death. The time series CRs being generally in the same direction as the bridge coding CRs validates our method.

The main advantage above bridge coding methods and the approach by Vallin and Meslé (1988) is that our results are corrected for coincidental time dependent fluctuations and not based on the distribution of causes of death in 1 year as in bridge coding methods. By conducting time series analysis we took into account the volatility of time series of causes of death (Anderson 2011). Because of yearly fluctuations, the double coding CRs for a particular ICD-transition determined in the last year before the transition will differ from the one determined in the first year after the transition. In our time series formulation, the CR is based on the number of deaths corrected for the time dependent fluctuations, instead of the real counted number.

This latter issue, combined with the possibility of non-representativeness of the analysed records in the sample in bridge coding (often the most difficult records can not be analysed automatically) might explain that although the time series CRs were generally in the same direction as the bridge coding CRs for Canada and Italy, differences in the magnitude were observed.

Additional advantage of the applied time series software is that the accuracy of the shift can be provided. For the ICD9–ICD10 revision in Italy, Canada and the Netherlands, we observed respectively 13, 7 and 4 significant cause-specific transitions among the 17 groups of causes of death. Because the confidence intervals around the yearly fluctuations are incorporated in the accuracy, these numbers tend to be lower compared to other studies not taking into account these fluctuations. The differences in significant transitions between the countries can have several causes, but an important one is the summed absolute magnitude of the individual interventions being for Italy and Canada twice the value for the Netherlands.

Additional advantages of our time series approach are that missing data can be interpolated, earlier ICD revisions can be bridged as well, and uniform implementation in different countries is possible.

An important property of our method is that it does not take into account medical content, except in the construction of the concordance table. It thus requires very limited information. For instance a transformation matrix, as in the approach by Vallin and Meslé (1988), Meslé and Vallin (1996), is not required. A direct consequence is that our results provide no information on where deaths end up that are removed from a particular cause and vice versa. Another disadvantage is that our method does not take into account the likelihood of exchange between different cause of death groups based on their medical content.

In addition, the general disadvantages of time series analysis apply to our method (see for example Chatfield 2004). Some important attributes need to be mentioned. First, due to the assumption of a smooth trend over time the method cannot distinguish true abrupt changes in the trend and abrupt data production changes (Rey et al. 2011). If a true change in death numbers occurs due to for example a medical intervention in the transition year this is being seen as a coding change. Second, data over a long period of time is needed before and after the coding change for the accurate estimation of the jump. Adding or removing a year to the time series will result in a slightly different solution. Dividing a causes of death in two causes of deaths or adding two causes of deaths to one, will also result in slightly different results. And finally, different time series method will give slightly different results due to different ways to divide the trend in its components.

Whereas previous regression methods applied their time series analysis to the log mortality rate, we have applied it to cause-proportional mortality. Using the log mortality rate is more accurate when applying the results directly for the study of cause-specific mortality trends. However, when the goal is to redistribute the cause-specific death numbers in a year (which can subsequently be used to assess cause-specific mortality trends accurately) cause-proportional mortality is to be preferred because of the additivity of the time series and its components. Note that non-significant breaks also need to be taken into account in the latter approach.

Just as with the double coding approach, our time series approach results in a classification change for 1 year, and it should not automatically be regarded as a correction for the entire time series. That is, the classification change is modelled as a constant intervention in Eq. 1. In other words, the ICD-transition is obtained as a constant number per cause of death in a certain year. Simple mathematics demonstrates that the uncertainty grows rapidly for this number as a function of stdev(*b*_*j*_) when the time till or from the transition increases. Moreover, our additive model does not take into account changes in the cause of death distribution over time. The validity of the obtained classification change is therefore limited to only a few years around the classification change and not optimal in terms of reconstructing very long time series. Using a multiplicative model instead would lead to a correction being approximately proportional to cause-proportional mortality, but still the use of a constant intervention can be questioned. The use of a time dependent intervention, based on the inclusion of additional demographic and/or medical information could be considered a useful alternative. An additional issue to take into account in the actual reconstruction of time series is the heterogeneity by sex and age group, as Rey et al. (2011) also suggested.

In this paper, as a case study, we applied our approach to the ICD9–ICD10 revision in three countries at Chapter level. We did not take into account potential intermediate coding changes caused for example by updates to ICD-9 and ICD-10 which generally only have a minor effect.

Next to the application to earlier ICD revisions, however, the method can be extended as well with an automatic detection method for breaks in order to find these incidental coding changes (Harvey and Koopman 1992) or abrupt data production changes like the move from manual to automated coding (Rey et al. 2011). Either years in which a change is likely to occur can be selected a priori or the methodology can detect breaks in the time series which a posterior need to be validated. In both cases additional subjective information from coders and data producers is crucial. The method can also be applied to a more detailed distinction of causes of death. It should be noted though that by including additional interventions and additional time series the calculation time increases fast because of the constraint. Applying the method to for instance the 65 causes of death of the European shortlist results in a calculation time of 24 h on an ordinary PC. Including sub-aggregates of causes of deaths in the calculation is necessary to obtain accurate results for them.

Moreover, it should be noted that for rare causes of death, which are more frequent in detailed classifications of death, a Poisson distribution would be more valid than the Gaussian distribution which was used in our time series model.

### Explanation of the Observed Results

Comparing the CRs for the ICD9–ICD10 transition estimated by time series analysis with the existing bridge coding CRs in Canada and Italy showed equal directions but differences in magnitude. This difference could be an artefact of the different ways the CRs are being calculated. That is, for the bridge coding CR the death numbers *x*_*j,t*_ are used, whereas for the time series CR we used the trend *y*_*j,t*_. Additional analysis in which we calculated the time series CRs using *x*_*j,t*_ showed roughly similar difference between the bridge coding CRs and the time series CRs. Another explanation for the difference—besides the difference in approach—is that both Italy and Canada do not use all records in their bridge coding. The records left out are likely to be selective, the most difficult to code, and therefore could influence the bridge coding CR.

Comparing the time series CRs for Italy in 2003, Canada in 1999 and the Netherlands in 1995 (Fig. 4) showed some interesting commonalities and differences.

Some of the differences could be explained by differences between the countries in the codes used for some cause of death chapters for ICD-9 (see Table 2). This might have affected the international comparison of time series CRs for Chapter I (Certain infectious diseases and parasitic diseases), Chapter III (Diseases of the blood and blood-forming organs and certain disorders involving the immune mechanism), Chapter IV: Endocrine, nutritional and metabolic diseases and Chapter XVI (certain conditions originating in the perinatal period).

Another possible explanation is differences in the implementation of the coding rules in the respective countries. For the Netherlands only manual coding is used, for Canada a mix of automatic and manual coding, and for Italy only automatic coding (see Table 1). This might partly explain why for the Netherlands the CRs were most often opposite to those in the other two countries (*N* = 4).

Also, the occurrence of the change from ICD9 to ICD10 in different years in the different countries, and therefore the CRs calculated for different years, might lead to small differences in the CRs.

In addition, differences between countries in cause of death certification and cause of death distribution may result in different CRs for the different countries (Anderson et al. 2001).

The larger CRs for Italy as compared to the Netherlands and Canada could have several explanations. First, Italy used a different methodology for automatic coding in ICD10 as compared to ICD 9 (ISTAT 2011). Second, it could be partly an artefact of applying our approach in Italy to a time series of only 5 years of data after the coding change including 2 years with missing information. The CRs for the double coding in Italy tend to indeed be smaller than the time series CRs for Italy. When comparing the double coding CRs between Italy and Canada more similar values showed.

Note that previous research for comparing CRs between countries used the results of different bridge coding studies, all with different implementations (Geran et al. 2005; Pace et al. 2007; ISTAT 2011). We, however, used the exact same software implementation of the time series method, which therefore can no longer affect the comparison and consequently can increase the comparability of CRs between countries.

The CRs in all three countries were strongly positive for diseases of the nervous system, diseases of the musculoskeletal system (not NL), and diseases related to pregnancy, childbirth and the puerperium and clearly negative for mental and behavioural disorders.

For diseases related to pregnancy, childbirth and the puerperium, the large CRs seem to be due to the small death proportions (<0.01 %) (see Table 2). The CR being especially large for Italy, followed by the Netherlands and then Canada strengthens this possible explanation. A possible explanation for Italy is that also several changes were implemented in the coding method with the transition from ICD9 to ICD 10 (ISTAT 2011).

For the remaining causes of death the consistently high or low CRs can be related to some of the main changes between ICD9 and ICD10 (ONS 2012a). For example, both the high CR for diseases of the nervous system and the high CR for diseases of the musculoskeletal system, which were observed in the UK as well, can be assigned to the application of Rule 3, which allows a condition which is reported in either Part I or II of the death certificate to take precedence over the condition selected using the other coding rules if it is obviously a direct consequence of that condition. In ICD-10 the list of conditions affected by Rule 3 is more clearly defined than in ICD-9 and is also broader in scope (ONS 2012a, b, c).

### Overall Conclusion

The methodology presented in this paper for bridging coding changes in causes of death has clear advantages over previous methods. Most importantly, our method obtains a consistent solution across causes of death. A factor which has largely been ignored in previous time series studies. In addition, the main advantage above the remaining methods is that our results are corrected for coincidental time dependent fluctuations and not based on the distribution of causes of death in 1 year with its likely coincidences. Also, the method can be uniformly applied to other countries and to former ICD revisions, can take into account incidental coding changes and can be extended to a more detailed distinction of causes of death.

In our paper we clearly demonstrated the importance of the constraint, and the validity of our methodology in terms of the CRs.

Our method, however, takes into account medical content only to a limited extent, and its results can be crude. Moreover the method does not provide information on where deaths end up that are removed from one cause and vice versa.

A logical step forward would be to integrate medical content to a larger extent, for example by including likely exchanges between causes of death based on the medical definition of ICD items.
